# Persistent Refractory Headaches Following Non-alcoholic Wernicke’s Encephalopathy in a Teenager

**DOI:** 10.7759/cureus.98878

**Published:** 2025-12-10

**Authors:** Irum Hina, Ehtesham Khalid

**Affiliations:** 1 Neurology, Medicare Hospital (Pvt) Ltd., Multan, PAK; 2 Neurology, Ochsner Medical Center, New Orleans, USA

**Keywords:** memory issues, persistent headache, thiamine deficinecy, weight loss, wernicke encephalopathy

## Abstract

Wernicke's encephalopathy (WE) is a neurological condition that presents with a clinical triad of ophthalmoparesis with nystagmus, confabulation, and ataxia. It is caused by thiamine deficiency (Vitamin B1), commonly associated with chronic alcoholism as well as non-alcoholic causes, and early diagnosis with prompt thiamine replacement is essential to prevent irreversible neurological damage. We report the case of a 17-year-old girl who lost 18 kg in one month due to aggressive dietary control without appropriate nutritional support and developed neurological signs that were consistent with WE. A clinical diagnosis of WE was made, and the patient was treated with intravenous and oral thiamine. She showed significant improvement in neurological symptoms, but also developed a new, persistent, refractory headache, which remained unresolved despite extensive pharmacologic and interventional headache treatments. Persistent headaches in the post-WE setting, as seen in this case, may represent a novel or underreported sequela that requires further research and clinical awareness.

## Introduction

Wernicke's encephalopathy (WE) is an acute or sub-acute neurological condition characterized by a triad of ophthalmoparesis with nystagmus, confabulation, and ataxia. It is caused by thiamine (vitamin B1) deficiency, which affects both the central and peripheral nervous systems by damaging the Papez circuit, particularly the thalamus, as well as peripheral nerves [1]. While alcohol-related causes are well-known, non-alcoholic causes are multiple and require prompt recognition and treatment to prevent long-term disability and morbidity. These include prolonged starvation, protein-energy malnutrition [2], malabsorption syndromes (e.g., celiac sprue), congenital transketolase deficiency, gastric bypass surgery, hyperemesis gravidarum, and even cases involving healthy infants fed with thiamine-deficient formulas [3].

Under-recognition of WE is common, and autopsy studies in the United States indicate that the condition is frequently missed during life. Brainstem lesions typical of WE on autopsy show an incidence ranging from 0.2% to 2.8%, and as high as 12.5% in the alcoholic population [4]. The male-to-female incidence ratio is 1.7:1. In terms of prognosis, WE is a potentially disabling and lethal condition. However, early treatment with thiamine can lead to rapid improvement in ataxia, ophthalmoparesis, and confusion. In contrast, memory and learning impairments tend to respond more slowly and may remain incomplete. Some patients who survive WE may go on to develop Wernicke-Korsakoff syndrome, while only about 20% recover fully. The diagnosis of WE is primarily clinical, and a positive response to thiamine therapy is often considered the best diagnostic indicator [5].

## Case presentation

A 17-year-old girl intentionally lost 18 kilograms in one month after her physician prescribed a local fiber supplement named “Ultra Special Fiber” for weight loss. The supplement was intended to be used in conjunction with a structured diet plan, which she was unable to follow due to severe nausea and anorexia. Over the next month, her condition progressively worsened, and she began experiencing multiple episodes of vomiting per day. She was taken to the emergency room (ER), where her condition temporarily improved after receiving intravenous supportive treatment.

**Figure 1:**
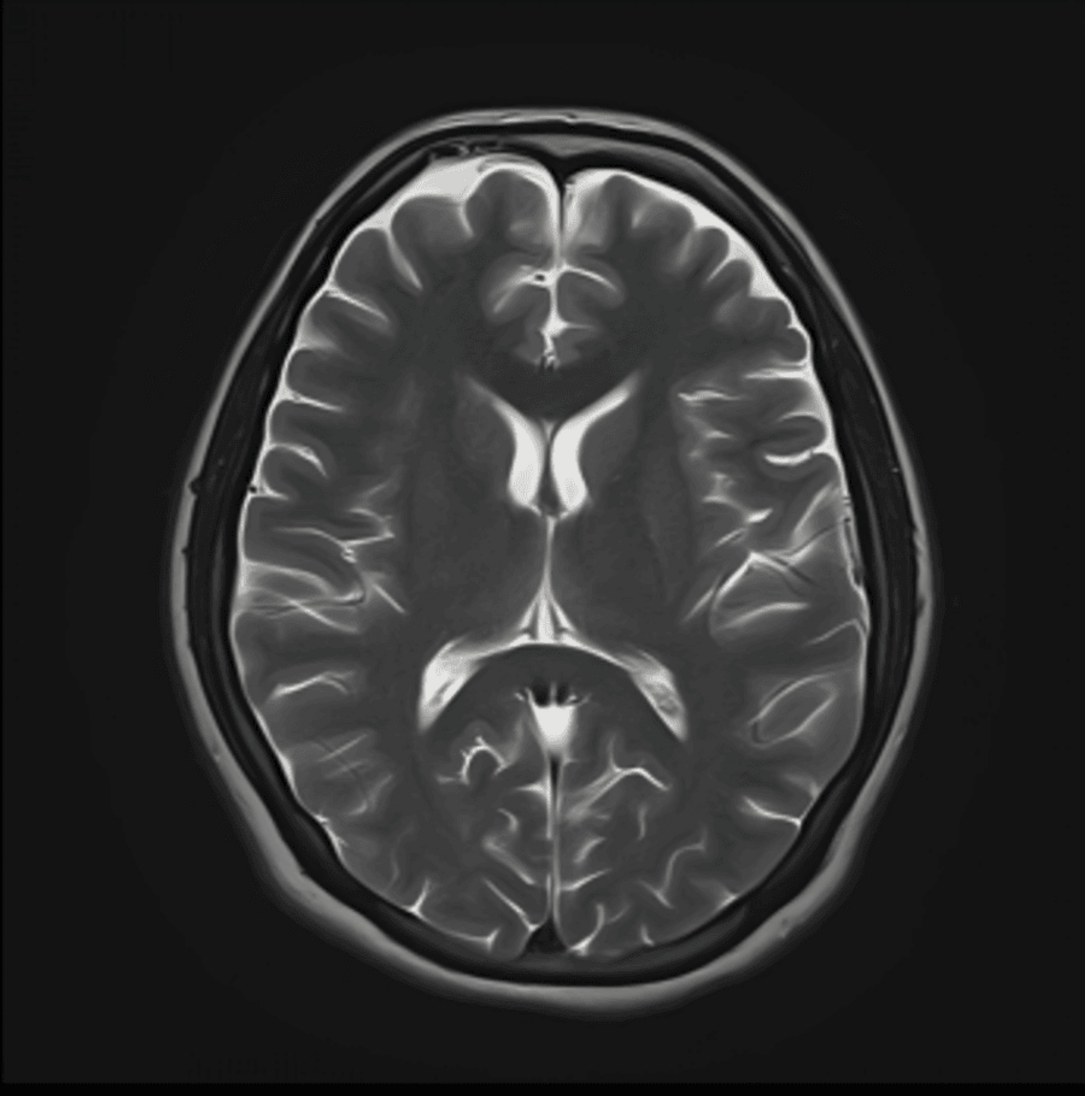
Normal axial T2-weighted magnetic resonance image (MRI) of the brain without contrast

**Figure 2:**
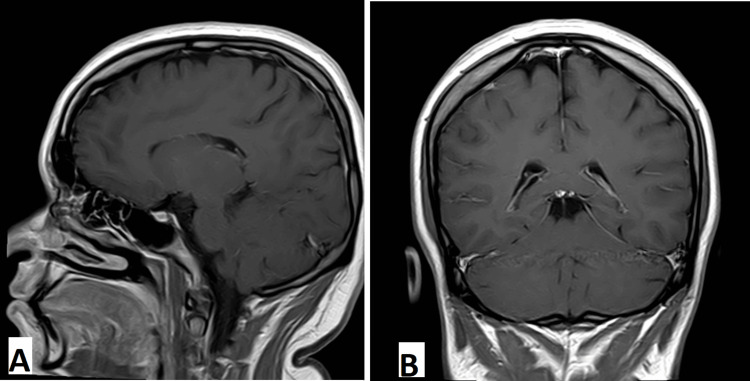
Normal T2-weighted MRI of the brain with contrast in sagittal view (A) and coronal view (B)

**Figure 3:**
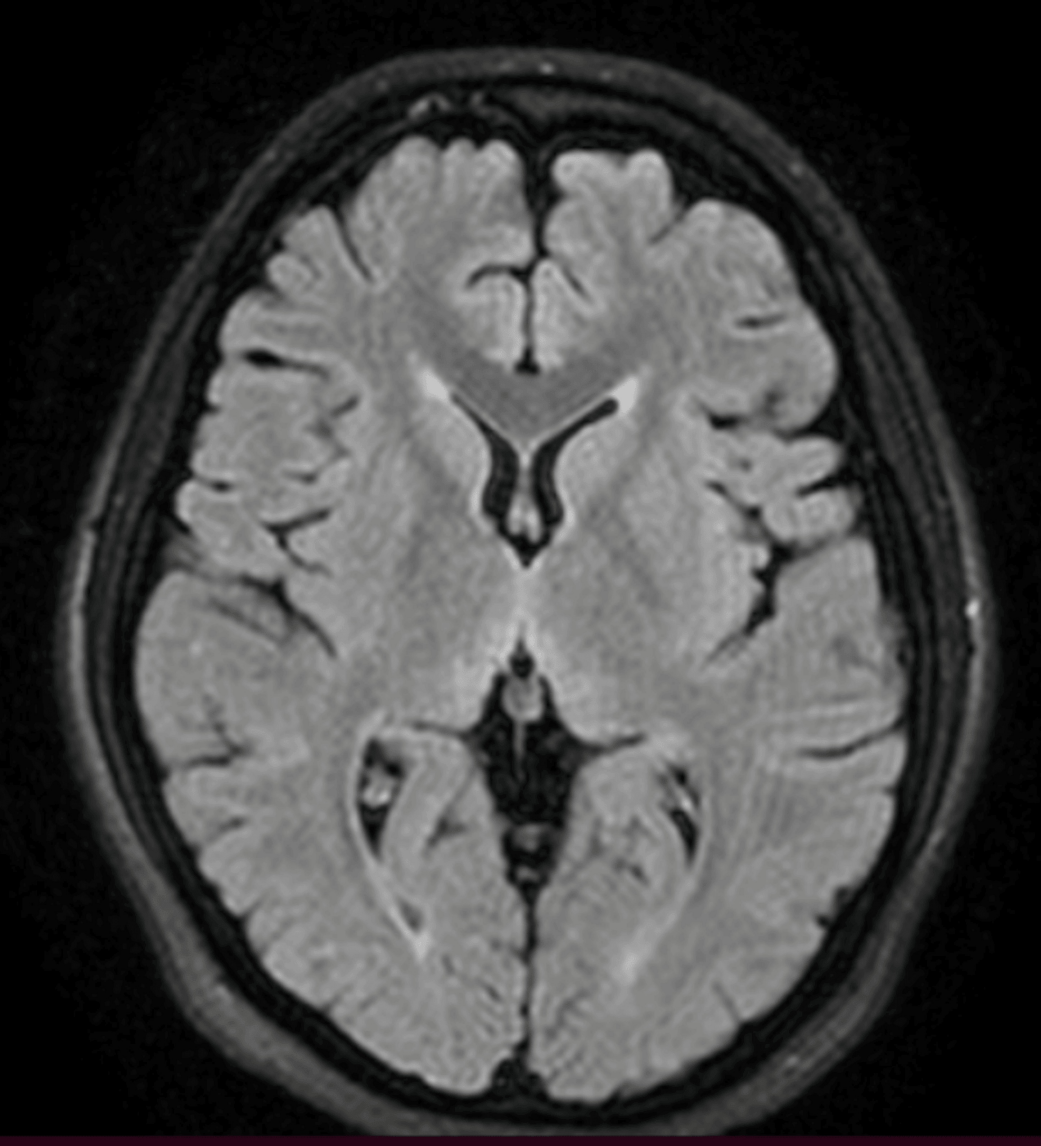
Axial T2 Fluid-Attenuated Inversion Recovery (FLAIR) MRI images of the brain show normal signal intensity throughout, with no evidence of abnormal hyperintensities or lesions

**Figure 4:**
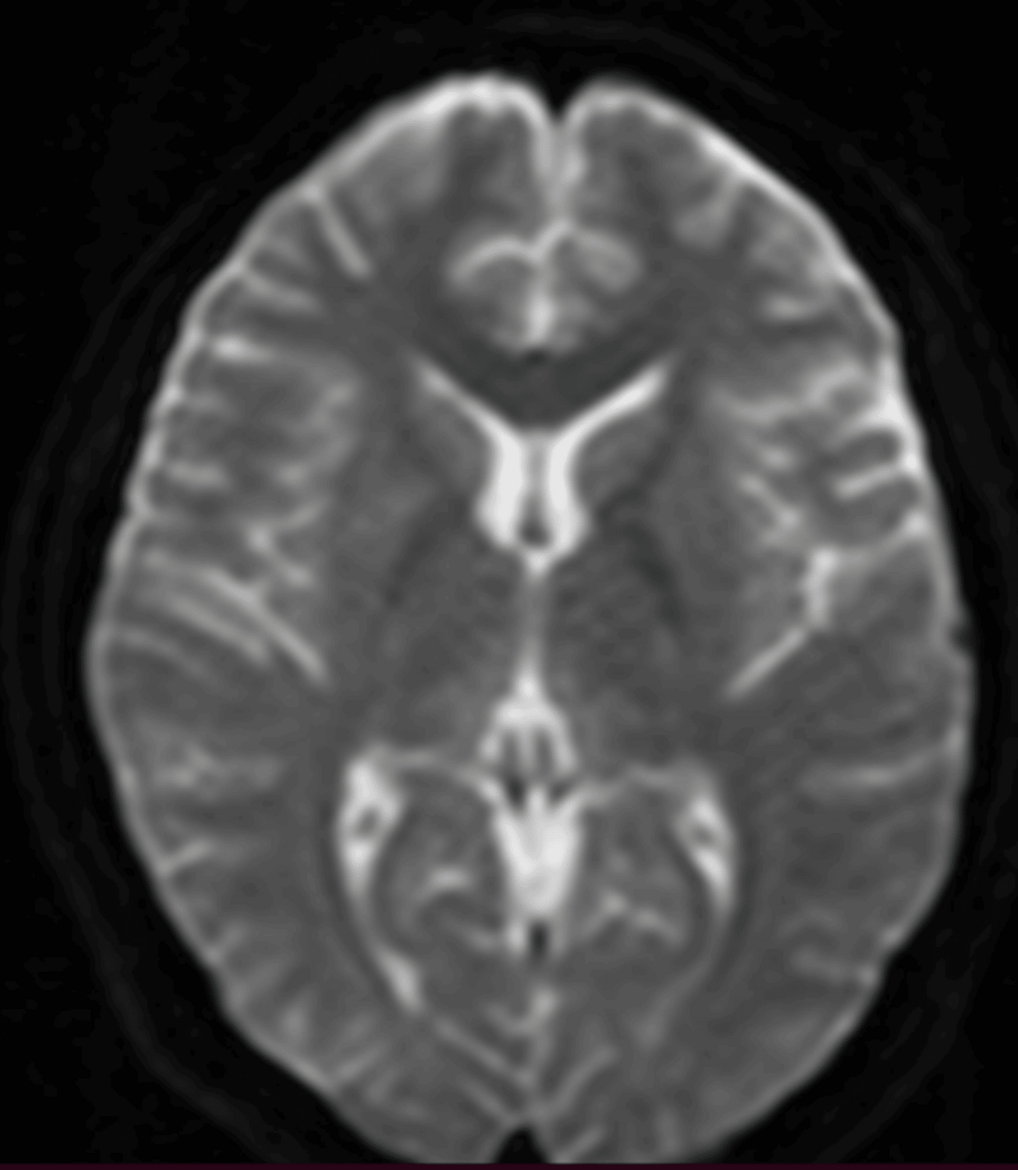
MRI of the brain, diffusion-weighted imaging (DWI), demonstrates no restricted diffusion

However, in the following days, her family observed new symptoms, including memory deficits and difficulty with walking and squatting. She also reported muscle cramps, knee pain, double vision, and mild breathing difficulty. The patient was brought to the emergency room again, where she was found to have gastritis on antral biopsy and acute cholecystitis on abdominal ultrasonography. Magnetic resonance imaging (MRI) of the brain (1.5 Tesla), both with and without contrast, was unremarkable with no findings typical of WE (Figures 1-4).

Additional laboratory investigations were also performed, as summarized below (Table 1).

**Table 1:** Laboratory work-up performed at the hospital

Test/Parameter	Result	Normal range
Alanine transaminase (ALT)	412 U/L	12 to 26 U/L
Serum prolactin	59.8 ng/mL	5 to 20 ng/mL
Serum sodium	142 mEq/L	135 and 145 mEq/L
Serum potassium	4.0 mEq/L	3.5 to 5.0 mEq/L
Hemoglobin A1c (HbA1c)	6.1%	Normal: below 5.7%, Pre-diabetes: 5.4%-6.3%, Diabetes: 6.4% and above
Follicle stimulating hormone (FSH)	5.85 IU/L	3.5 to 12.5 IU/L
Luteinizing hormone (LH)	4.49 IU/L	2.4 to 12.6 IU/L
Thyroid stimulating hormone (TSH)	2.24 µIU/mL	0.4-4.0 µIU/mL
Total tetraiodothyronine (T4)	9.6 µg/dL	5–12 µg/dL
C-Reactive protein (CRP)	0.5 mg/L	0.3–1.0 mg/dL
Erythrocyte sedimentation rate (ESR)	11 mm/hr	0 to 15 mm/hr
Serum creatinine	0.7 mg/dL	0.5 to 1.0 mg/dL
Creatine phosphokinase (CPK)	183 U/L	48 to 200 U/L
Tissue transglutaminase antibody	1.1 U/mL	Less than 4.0 U/mL

No thiamine (Vitamin B1) supplementation was initiated initially, although her acute condition improved with the symptomatic treatments provided at that time. Over the following two months, she began to experience headaches with a pressure-like sensation around her left eye, along with some sensitivity to light and sound, but without any gastrointestinal symptoms. It was during this period that she presented to our clinic for a second opinion regarding her diagnosis.

Neurological examination revealed horizontal gaze-evoked nystagmus with the fast component directed to the left and the slow component to the right, as well as vertical nystagmus with the fast component in the upward direction (Video 1).

**Video 1:** Video showing horizontal gaze-evoked nystagmus with the fast component directed to the left and the slow component to the right, as well as vertical nystagmus with the fast component in the upward direction

Additional findings included impaired memory, with a Montreal cognitive assessment (MoCA) score of 19/30; diminished vibration sensation up to the ankles; absent knee reflexes and reduced ankle reflexes; bilateral extensor plantar responses; and difficulty performing tandem gait.

We strongly suspected WE and initiated intravenous thiamine as a stat dose, followed by daily oral supplementation with thiamine, methylcobalamin, and magnesium. The patient responded well to this treatment. After one month, she showed significant improvement in memory, diplopia, sensation, and proximal muscle strength. She was able to perform squats and tandem gait with only mild difficulty. Her MoCA scores improved to 29/30 on subsequent visits, although ocular findings and headaches persisted.

Previously, prior to the onset of her illness, the patient had no history of intermittent headaches. However, over the next six months after starting oral thiamine, her headaches persisted, though they were mild in intensity, occurring only two to three times per month. After the seventh month, the headaches began to worsen, presenting as diffuse pain radiating to the neck with an intensity of 8/10 on the pain scale. She experienced multiple brief episodes per day, each lasting five to 10 minutes, along with a mild baseline headache. Oral gabapentin and magnesium were found to exacerbate her headaches and were therefore discontinued. Despite multiple oral and intravenous medications administered according to standard protocols, including intravenous migraine cocktail and botulinum toxin therapy targeting the scalp muscles, her headaches did not improve. Other medications trialed for headache management included venlafaxine, propranolol, coenzyme Q10, escitalopram, baclofen, lisinopril, and verapamil, along with occasional rescue medications for breakthrough pain.

Further interventions, including bilateral sphenopalatine ganglion blocks, and occipital nerve blocks, were also attempted without benefit. To date, she continues to experience ocular abnormalities (nystagmus) and persistent daily headaches, characterized by 12-14 brief episodes of sharp pain each day, in addition to a constant baseline headache, ongoing for the past four years. However, she has been able to continue her studies without any cognitive or functional impairment.

## Discussion

WE is an acute neurological condition that, if left untreated or not managed promptly, can progress to the chronic and irreversible Wernicke-Korsakoff syndrome. Therefore, early recognition and timely treatment are crucial, as they can significantly improve patient outcomes [6]. The diagnosis of WE is primarily clinical, based on hallmark findings such as ocular abnormalities, memory impairment, and gait disturbance, supported by basic laboratory investigations.

Although serum thiamine levels can assist in diagnosis, these tests are often unavailable in developing countries. Neuroimaging, particularly magnetic resonance imaging (MRI), is commonly used to exclude alternative diagnoses such as tumors or stroke. However, no single diagnostic modality offers high sensitivity or specificity for WE. In our patient, serum thiamine levels were not available, and MRI findings were unremarkable. Nonetheless, the patient exhibited the classic clinical triad of WE, which strongly supported the diagnosis.

Interestingly, persistent or refractory headaches are not commonly described following recovery from acute WE. A recent study suggests that migraine-associated nausea and anorexia may contribute to mild-to-moderate thiamine deficiency, which can, in turn, worsen gastrointestinal symptoms in a cyclical pattern. This may increase the chronicity and frequency of headache or migraine attacks [7]. Breaking this cycle through thiamine supplementation has shown promise as a therapeutic strategy in patients with chronic migraine.

In this case, the patient had no prior history of headaches or migraines, raising the possibility that her new-onset, persistent, and refractory headaches may represent a previously underrecognized post-WE complication. Although the precise pathophysiological mechanism remains unclear, this observation warrants further investigation, particularly in non-alcoholic WE patients who present with atypical post-recovery symptoms. We hypothesize that acute WE may predispose survivors, especially young individuals experiencing rapid weight loss, to long-term, treatment-resistant headache disorders, potentially due to persistent mitochondrial dysfunction or impaired thiamine transport.

Radiographic imaging remains a useful adjunct in the early diagnosis of WE. A 2008 study involving over 56 patients with WE demonstrated distinct differences in MRI findings between alcoholic and non-alcoholic patients. Alcoholic patients more frequently exhibited contrast enhancement of the mammillary bodies and thalami [8]. Another study reported that symmetrical T2-weighted and fluid-attenuated inversion recovery (FLAIR) hyperintensities in the thalami, mammillary bodies, tectal plate, and periaqueductal region are typical in alcoholic WE, whereas atypical regions such as the cerebellum, cranial nerve nuclei, and cerebral cortex are more frequently involved in non-alcoholic cases [9].

In our patient, MRI findings were normal, with no classical or atypical radiological features of WE. This further underscores the importance of clinical judgment in diagnosing WE, particularly in non-alcoholic individuals who may present with subtle or atypical signs.

## Conclusions

This case highlights the importance of recognizing non-alcoholic causes of WE, particularly in young individuals who engage in extreme dietary restrictions in pursuit of physical fitness goals. Despite the absence of classical radiological findings and an initial delay in thiamine supplementation, the clinical diagnosis of WE was strongly supported by the characteristic triad of ophthalmoparesis with nystagmus, ataxia, and cognitive impairment. Early and aggressive thiamine replacement resulted in significant neurological improvement, effectively preventing long-term cognitive or functional disability.

Notably, the emergence of a new, persistent, and refractory headache, despite the resolution of other symptoms, raises the possibility of an under-recognized post-WE complication that warrants further investigation. This case underscores the need for heightened clinical suspicion of WE in patients with malnutrition or prolonged vomiting, irrespective of alcohol use history, and reinforces the role of early thiamine administration as a low-risk, potentially life-saving intervention.
